# Hospital Managers in Municipal Office

**Published:** 1916-11-18

**Authors:** 


### HOSPITAL MANAGERS IN MUNICIPAL OFFICE.

At the beginning of another municipal year we may well recall the proofs which have often been given of the advantages which accrue from medical men and hospital managers engaging in municipal life. The difficulty is for such men to find the necessary time, for the post of mayor is, in the vulgar sense, a thankless one. There is more service than prestige attached to it, and only those deep enough to know that greatness means service, not domination, realise that the post of labour is the post of honour. A mayor has to give his time to much necessary but dull work (that absorbed by attendance at innumerable meetings is by itself a big sacrifice to a busy man), and " the ha'pence " associated with the work mostly have to come out of his own pocket, while the kicks of criticism are always about his path. Yet the medical man and the hospital manager make some of the most useful of mayors. The knowledge of municipal conditions, of the needs of the people and the borough, are given by a hospital training as in no other way. We say this confidently, for the hospital man is trained to estimate the need before the cost. His conscience is the least corrupted by commercial considerations. That is a great gain, and illustration of the value of such a man in this office has again been provided at Leeds, where Mr. Charles Lupton, of the Leeds General Infirmary, has been able as Lord Mayor to perform work of unrivalled value both as regards securing the future of the newly extended General Infirmary, and in regard to the great expansion of hospital accommodation for the wounded recently occupying the attention of the city. Our own columns have been a file of his difficulties, efforts, and successes, which were lately crowned by a remarkable personal tribute from Field-Marshal Lord French on his recent visit to the works and hospitals of the city.

Now we venture to repeat that, personality apart, such successes in municipal work are largely assured when hospital officers and medical men can be persuaded to shoulder the burden of office. We also assert with equal confidence that such successes are not dependent on the war, which has pushed hospital affairs so far into the foreground. The influence of the war in hospital, as in other directions, will not pass away with the passing of the Prussian hierarchy. Hospital problems will maintain an enhanced importance after the war. The many hundreds of beds in civil hospitals at present allocated to the wounded will be claimed for the army of civilians now swelling the waiting lists. These lists are not everywhere swollen in equal proportions, and on the whole the marvel is that they have been kept within manageable dimensions at all. Still, long waiting lists do exist, and until the war is over there is not much chance of reducing them. This problem of beds will become one of the most burning of local questions after the war, and who so capable of solving it as hospital managers and medical men if entrusted with municipal office? As Leeds and other cities have shown, the mayors of the future should include a far larger number of hospital lay and medical managers.

